# Linear and non-linear dependencies between copy number aberrations and mRNA expression reveal distinct molecular pathways in breast cancer

**DOI:** 10.1186/1471-2105-12-197

**Published:** 2011-05-24

**Authors:** Hiroko K Solvang, Ole Christian Lingjærde, Arnoldo Frigessi, Anne-Lise Børresen-Dale, Vessela N Kristensen

**Affiliations:** 1 Department of Genetics, Institute for Cancer Research, Oslo University Hospital, Radiumhospitalet, Montebello, 0310 Oslo, Norway; 2 Department of Biostatistics, Institute of Basic Medical Science, University of Oslo, Norway; 3 Biomedical Research Group, Department of Informatics, University of Oslo, Norway; 4 Statistics for Innovation - (sfi)2, NR -Norwegian Computing Centre, Norway; 5 Institute for Clinical Medicine, University of Oslo, Norway; 6 Institute for Clinical Epidemiology and Molecular Biology (EpiGen), Faculty of Medicine, Division Akershus University Hospital, University of Oslo, Norway

## Abstract

**Background:**

Elucidating the exact relationship between gene copy number and expression would enable identification of regulatory mechanisms of abnormal gene expression and biological pathways of regulation. Most current approaches either depend on linear correlation or on nonparametric tests of association that are insensitive to the exact shape of the relationship. Based on knowledge of enzyme kinetics and gene regulation, we would expect the functional shape of the relationship to be gene dependent and to be related to the gene regulatory mechanisms involved. Here, we propose a statistical approach to investigate and distinguish between linear and nonlinear dependences between DNA copy number alteration and mRNA expression.

**Results:**

We applied the proposed method to DNA copy numbers derived from Illumina 109 K SNP-CGH arrays (using the log R values) and expression data from Agilent 44 K mRNA arrays, focusing on commonly aberrated genomic loci in a collection of 102 breast tumors. Regression analysis was used to identify the type of relationship (linear or nonlinear), and subsequent pathway analysis revealed that genes displaying a linear relationship were overall associated with substantially different biological processes than genes displaying a nonlinear relationship. In the group of genes with a linear relationship, we found significant association to canonical pathways, including purine and pyrimidine metabolism (for both deletions and amplifications) as well as estrogen metabolism (linear amplification) and BRCA-related response to damage (linear deletion). In the group of genes displaying a nonlinear relationship, the top canonical pathways were specific pathways like PTEN and PI13K/AKT (nonlinear amplification) and Wnt(B) and IL-2 signalling (nonlinear deletion). Both amplifications and deletions pointed to the same affected pathways and identified cancer as the top significant disease and cell cycle, cell signaling and cellular development as significant networks.

**Conclusions:**

This paper presents a novel approach to assessing the validity of the dependence of expression data on copy number data, and this approach may help in identifying the drivers of carcinogenesis.

## Background

Cancer development and progression are complex processes involving a series of genetic and functional abnormalities. Joint analysis of array comparative genomic hybridization (aCGH) copy number data and microarray gene expression data may uncover biological relationships relevant to our understanding of cancer. Previous whole-genome analyses of copy number and gene expression have led to the identification of global cellular processes underlying malignant transformation and progression. In addition to basic biological applications, clinical challenges such as early diagnosis, risk stratification, and treatment failure [1-3] have been addressed. Combining large-scale data from a variety of analyses of tumors at the DNA, RNA and protein levels has resulted in novel areas of research directed toward better defining the molecular basis of malignancy [4,5].

Many studies address whether and to what extent gene expression alterations correlate with chromosomal abnormalities. An early study of Hyman et al. [6] on breast cancer cell lines using aCGH reported that 44% of the highly amplified genes were over-expressed and 10.5% of the highly over-expressed genes were amplified. These genes include known oncogenes and potential therapeutic targets. Another early aCGH study on breast cancers [7] found that 62% of the highly amplified genes showed moderately or highly elevated expression. DNA copy number was found to influence gene expression across a wide range of DNA copy number alterations (deletion and low-, mid- and high-level amplification), and a two-fold change in DNA copy number lead on the average to a 1.5-fold change in the mRNA level. Overall, at least 12% of all variation in gene expression among breast tumors was directly attributed to variation in gene copy number. Permutation analysis identified genes whose expression was systematically attributable to gene amplification. Several other association studies also consider a linear correlation structure [8-10]. Van Wieringer et al. [11] developed a nonparametric test to detect copy number induced differential gene expression, while Schäfer et at. [12] proposed a bivariate assessment to find DNA regions of equally directed abnormalities in copy number and gene expression. A recent study of Turner et al. [13] on triple negative breast cancers identified genes consistently over-expressed when amplified. The above attempts to quantify the amount of RNA that may result from an aberrant DNA locus applied a linear regression to model the dependence of the expression data on the copy number data.

Given the fact that correlations between copy number and expression are used to estimate drivers in carcinogenesis [14] it is very important to assess the validity of the chosen methods in establishing the form of such dependences. Most methods employ linear regression, but the biological reality is that this interaction is readily derailed from linearity by a number of regulatory mechanisms contributing to gene expression, such as transcriptional activation, miRNA-driven regulation, and DNA methylation, to mention a few. The possibility of a nonlinear relationship between copy number alterations and the expression of specific genes must therefore be explored and related to the mechanism of deregulation of gene expression in the tumor. We believe that the identification of nonlinear relationships will enable us to identify the regulatory mechanisms of abnormal mRNA expressions of relevance in the cancer process. Nonlinearity may reflect biological pathways of regulation, such as DNA methylation, histone modification and micro RNA, as well as the presence of transcription binding and miRNA target sites, enhancers and insulators; the presence of these factors must surely influence the relationship to mRNA expression beyond copy number itself.

In mathematical terms, any smooth nonlinear function can be approximated locally by a quadratic polynomial. In line with this, we consider a model in which mRNA expression is a quadratic function of copy number to investigate the presence of nonlinearity. We classify each locus according to whether the quadratic model provides a worse, equal or better fit to the data than the linear model. The genes for which the copy number/mRNA associations were classified as being linear or nonlinear were further analyzed by pathway analysis in order to discuss their biological interaction and regulatory mechanisms. We apply the procedure to copy number data (llumina 109 K SNP array) and expression data (Agilent 44 K expression array) from 102 breast carcinoma samples.

## Results and Discussions

### Spearman correlation

**Figure 1:**
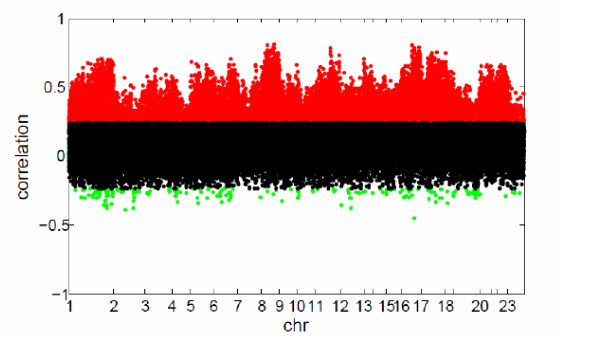
**In-cis correlations between CGH an mRNA**. Number of significant correlated parts were in about 13 K loci (FDR 5%). Horizontal axis indicates chromosomes and vertical axis indicates Spearman correlation values (significant positive (red), negative (green) and uncorrelated (black)).

As a preliminary investigation, we first calculated the *in-cis *Spearman correlation between copy number and expression for all 40 K genomic locations (see Method). Significant positive and negative correlations after false discovery rate (FDR) correction were detected, resulting in about 16 K associated pairs (Figure 1). Specifically, the number of significantly correlated pairs was 15,654 at FDR10%, 13,085 for FDR5% (Figure 1), and 9,294 for FDR1%; the vast majority being positive correlation as expected, since a deletion most likely would lead to a decrease in mRNA expression and an amplification to increased mRNA expression. Still, some negatively correlated loci were seen, which may simply indicate noise or, on the other hand, deserve more detailed sequence analysis since they may be deletions of inhibitory elements or amplifications of negative regulators and thus reflect feedback mechanisms.

### Application of the linear and quadratic models

**Figure 2:**
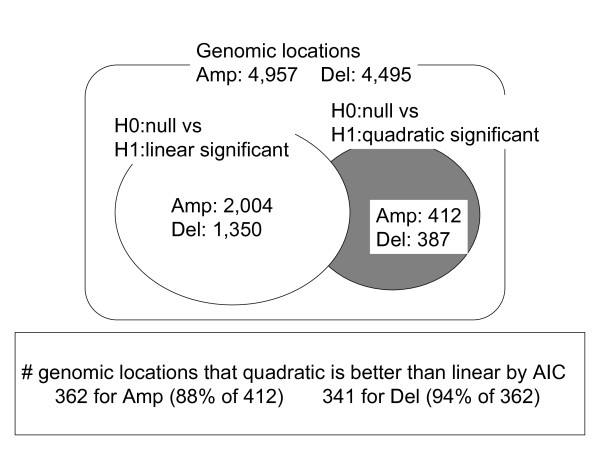
**Identified genomic locations by linear/quadratic models**.

**Figure 3:**
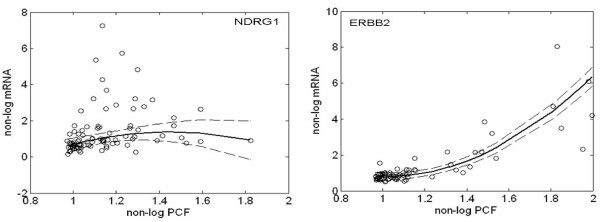
**Estimated predictions for NDRG1 and ERBB2 obtained by quadratic model**. Scatter plots are on vertical axis for non-logarithmic mRNA data vs. horizontal axis for non-logarithmic PCF logR. Estimated predictions and the 95% confidential interval are represented by solid and dashed lines, respectively.

For 4,957 and 4,495 gene locations for Amp and Del, respectively, we applied the proposed linear and quadratic models introduced in the Methods section. The numbers for a significant linear/quadratic relationship are summarized in Figure 2. A significant *linear term *according to model (1) was observed for 2,004 loci of the amplified genes (Amp) and 1,350 loci for the deleted genes (Del) based on FDR5%. A significant *quadratic term *according to model (2) was observed in 412 loci for Amp and 387 loci for Del. The overlapping parts such as loci with both significant linear and quadratic components were 263 for Amp and 215 for Del. These were also counted as loci significantly identified by the quadratic model. Genes identified by the quadratic model were amplified genes such as *NDRG1 *and *ERBB2*. Figure 3 displays the scatter plot of the data (circles), the predictions (solid line) and the 95% confidence intervals (dashed lines) by the non-linear model for these genes. The coefficient for the quadratic term of *NDRG1 *was negative, suggesting a mechanism for suppressing expression despite amplification; on the other hand, the positive coefficient for *ERBB2 *suggested the existence of further enhancement of expression. Consequently, the different shapes of non-linearity may suggest different molecular mechanisms of up-regulation and thus require further verification by functional studies. Indeed, this type of analysis should be considered as only indicating the chromosomal locations with copy number alterations, that is, where additional regulatory influences are most likely. Furthermore, this analysis can be obscured by the fact that there is a large inter-individual variation as a result of complex trans gene-gene regulation events in each tumor [15]. For instance, for *NDRG1 *there is clearly a population of samples where a higher amplification does not result in higher mRNA levels, but another population where this is the case.

### Accuracy for predictions identified by linear/quadratic model

**Figure 4:**
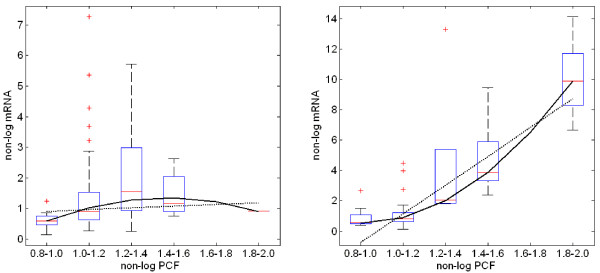
**Box-plots for the observations of NDRG1 and ERBB2 and predictions by linear and quadratic models**. The plots are illustrated for six regions to indicate the degree of dispersion of each particular region. Solid and dash-dotted lines indicate the predications by linear and quadratic models, respectively.

When trying to characterize the relation between copy numbers and expression levels, we need to filter out the very many observations which fall around normal CGH values: This cloud of data reduces the fit of any model, since it contains uninteresting individual variability attached to the vast majority of points. If we randomly thinned the clouds, we would balance the data better along their range and see relations more clearly. In order to do this, we performed median regression. We first divided the CGH data into five regions where a region is defined by *x_ij _*-1.0 in models (1) and (2) (see Methods). Then we created box plots for each of these 5 regions to indicate the degree of dispersion of each particular region. To the median of the data in each region, we again applied the linear and quadratic regression models (Figure 4). Solid and dash-dotted lines indicate the predictions by quadratic and linear models, respectively.

The variances of the prediction error for linear and quadratic models are 0.112 and 0.0281 for *NDRG1*, and 2.94 and 0.313 for *ERBB2*. Furthermore, AICs for the linear and quadratic models are 9.25 and 4.33 for *NDRG1*, and 25.6 and 16.4 for *ERBB2*, respectively. These results show that the quadratic model was better than the linear model.

The loci identified as significant by the *T*-test and partial *F*-test were also significant from the viewpoint of model selection, based on the AIC (see Additional file 1).

Finally, mRNA expression data involve multiple probes per gene in many cases, which allows us to check for consistency. In the gene lists identified as quadratic relationship for Amp, we found multiple probes with both negative and positive quadratic coefficients for several genes, such as *SDCCAG8, CLDN11 *and *STAU2*.

### Scatter plots for top 10 linear/quadratic models

When we investigate the individual scatter plots for CGH copy number vs. mRNA expression, we can observe in many cases a large population of samples with a relatively strong effect on the mRNA level. Even though they are found significant through model evaluation by AIC and statistical tests, some predictions of linear and quadratic relationships, as illustrated, are rather poor. As discussed above for the case of *NDRG1*, there is clearly a population of samples where a higher amplification does not result in higher mRNA levels, and another population where this is clearly the case. A multitude of hypotheses could explain this effect, for example the higher mRNA levels may not be copy-number-dependent, i.e. the copy number plays a small role here, or the copy number effect may be conditioned on another variable, and such a variable would differ from sample to sample. The scatter plots and the predictions of the top 10 models for each of the following categories are presented as follows: i) linear model with positive (first five panels) and negative (latter five panels) linear coefficients for Amp are shown in Additional file 2 Figure 2a; ii) linear model with positive and negative linear coefficients for Del - in Additional file 2 Figure 2b; iii) quadratic model with positive and negative quadratic coefficients for Amp - in Additional file 2 Figure 2c; and iv) quadratic model with positive and negative quadratic coefficients for Del - in Additional file 2 Figure 2d. The top 10 models were selected through the following two procedures: 1.We sorted p-values for the test statistics (see Methods), and 2. From the lowest p-values of test statistics, we selected the 5 lowest p-values of positive and of negative linear/quadratic coefficients.

In Additional file 2 Figures 2c and 2d, we show both linear and quadratic predictions by dotted and solid lines, respectively. The genomic locations, estimated linear/quadratic coefficients, the p-values for the coefficients and statistics, likelihood and AIC for linear/quadratic models are summarized in Additional file 3. As we can see in Additional file 2 Figures 2a and 2b, the scatter plots tend to distribute uniformly around the predictions. On the other hand, in the case of the quadratic model with positive quadratic coefficient (Additional file 2 Figure 2 c, first five panels, here called 'case 1'), a few data are distributed in higher expression levels of higher copy number regions. However, in the case of the quadratic model with negative coefficient for Amp (Additional file 2 Figure 2c, second five panels, called 'case 2'), we see that many samples are distributed in higher expression levels for normal copy regions, while some samples are distributed in lower expression levels for higher copy regions. Furthermore, in the case of the quadratic model with positive coefficient for Del (Additional file 2 Figure 2d, first five panels, called 'case 3'), a few samples showed higher expression in the loss region, and in the case of the quadratic model with negative coefficient for Del (Additional file 2 Figure 2d, second five panels, called 'case 4'), many samples showed higher expression in the normal copy region, while a few samples showed lower expression in the loss region. For the cases of 'case 2' and 'case 4', we can construct two different models to describe the obtained characteristics:1) Suppression of expression despite amplification, and 2) The linear relationship of expression level to copy number variability.

### Pathway analysis

To investigate the biological functional mechanisms, pathway analysis was performed by Ingenuity Pathway Analysis (IPA). We summarize the outputs for the associated network functions, diseases and disorders, and molecular and cellular functions in Table 1. The p-value associated with a biological process or pathway annotation is a measure of its statistical significance with respect to the Functions/Pathways/Lists Eligible molecules for the dataset and a Reference Set of molecules (which define the molecules that could possibly have been Functions/Pathways/Lists Eligible). The p-value was calculated with the right-tailed Fisher's Exact Test, and we applied the Benjamini-Hochberg [16] multiple-testing p-value. The ratio of the canonical pathways is calculated by dividing the number of molecules in a given pathway that meet the cut-off criteria by the total number of molecules that make up that pathway. Networks were scored based on the number of Network Eligible molecules they contained. In Table 1 a score over 10 was recognized as a meaningfully higher score. The network Score was based on the hypergeometric distribution (Source: IPA online manual).

**Table 1:** Summary for pathway analyses

models	Top Canonical Pathways	-log(B_H p-value)	Ratio
Linear Amp	Estrogen receptor signaling	6.95e-01	15/135 (0.111)
	
	Pyrimidine Metabolism	3.31e-01	15/151 (0.099)
	
	Mitochondrial Dysfunction	3.31e-01	13/127 (0.102)
	
	Molecular Mechanisms of Cancer	1.77e-01	25/359 (0.07)
	
	Oxidative Phosphorylation	1.77-01	13/140 (0.093)

Linear Del	Purine metabolism	1.1e00	23/303 (0.076)
	
	DNA Methylation and Transcriptional Repression Signaling	5.24e-01	4/23 (0.174)
	
	Reelin Signaling in Neurons	5.24e-01	8/82 (0.098)
	
	Pyrimidine Metabolism	5.24e-01	12/151 (0.079)
	
	Toll-like Receptor Signaling	5.24e-01	6/51 (0.118)

Quad Amp	P13K/AKT signaling	1.93e00	9/132 (0.068)
	
	Neuregulin signaling	1.49e00	7/98 (0.071)
	
	PTEN signaling	4.97e-01	6/120 (0.05)
	
	TR/RXR activation	2.95e-01	5/93 (0.054)
	
	ERK5 Signaling	2.74e-01	4/67 (0.06)

Quad Del	Cell cycle regulation by BTG family proteins	1.88e00	5/36 (0.139)
	
	CTLA4 signaling in cytotoxic T lymphocytes	1.83e00	7/98 (0.071)
	
	Ceramide signaling	1.48e00	6/85 (0.071)
	
	Wnt/ß-catenin signaling	6.97e-01	7/173 (0.04)
	
	IL-2 signaling	6.97e-01	4/59 (0.068)

models	Top Associated Network Functions		Score

Linear Amp	Embryonic Development, Tissue Development, DNA Replication, Recombination, and Repair		41
	
	Dermatological Diseases and Conditions, Genetic Disorder, Metabolic Disease		35
	
	Cell Signaling, RNA Post-Transcriptional Modification, Gene Expression		35
	
	Gene Expression, Cellular Compromise, Cardiovascular System Development and Function		35
	
	Cellular Development, Carbohydrate Metabolism, Lipid Metabolism		33

Linear Del	Cellular assembly and organization, Cellular function and maintenance, DNA replication, Recombination, and Repair		37
	
	RNA Post-Transcriptional Modification, Gene Expression, Immunological Disease		37
	
	Embryonic Development, Cell Morphology, Cellular Development		37
	
	Gene Expression, Cell Cycle, Cellular Development		33
	
	Cellular Movement, Nervous System Development and Function, Cellular Assembly and Organization		27

Quad Amp	Molecular Transport, Small Molecule Biochemistry, Cell Cycle		44
	
	Gene Expression, Cancer, Gastrointestinal Disease		41
	
	Cell Morphology, Cell-To-Cell Signaling and Interaction, Cellular Assembly and Organization		26
	
	Gene Expression, Endocrine System Disorders, Genetic Disorder		21
	
	Gene Expression, Connective Tissue Disorders, Immunological Disease		19

Quad Del	Cell Cycle, Carbohydrate Metabolism, Lipid Metabolism		45
	
	Organismal Injury and Abnormalities, Cellular Development, Cell Death		23
	
	Post-Translational Modification, Protein Degradation, Protein Synthesis		21
	
	Cellular Assembly and Organization, Nervous System Development and Function, Cell-To-Cell Signaling and Interact		21
	
	Cell-To-Cell Signaling and Interaction, Hematological System Development and Function, Immune Cell Trafficking		19

models	Diseases and disorders	B-H p-value	# Molecules

Linear Amp	Cancer	2.24e-05 - 4.45e-01	120
	
	Reproductive system disease	2.24e-05 - 4.39e-01	86
	
	Development disorder	6.55 e -02 - 4.39e-01	19
	
	Inflammatory response	3.77e-01 - 4.39e-01	5
	
	Genetic disorder	4.39e-01 - 4.39e-01	38

Linear Del	Cancer	4,27e-06 - 2.4e-01	102
	
	Reproductive system disease	4.27e-06 - 2.4e-01	75
	
	Infectious disease	6.9e-03 - 2.4e-01	58
	
	Infection mechanism	7.62e-03 - 2.4e-01	60
	
	Dermatological Diseases and Conditions	9.78e-02 - 2.4e-01	28

Quad Amp	Cancer	6.54e-03 - 1.68e-01	73
	
	Reproductive system disease	6.54e-03 - 1.68e-01	34
	
	Gastrointestinal disease	1.57e-01 - 1.68e-01	9
	
	Hepatic system disease	1.57e-01 - 1.68e-01	8
	
	Inflammatory disease	1.57e-01 - 1.68e-01	12

Quad Del	Genetic disorder	1.54e-01 - 2.11e-01	33
	
	Neurological disease	1.54e-01 - 2.11e-01	24
	
	Psychological disorders	1.54e-01 - 2.11e-01	18
	
	Cancer	1.54e-01 - 2.11e-01	15
	
	Metabolic Disease	1.54e-01 - 2.11e-01	7

models	Molecular and Cellular Functions	B-H p-value	# Molecules

Linear Amp	Gene expression	5.23e-03 - 4.45e-01	133
	
	Cell death	1.21e-01 - 4.45e-01	50
	
	Cellular growth and proliferation	1.21e-01 - 4.39e-01	33
	
	Molecular transport	3.77e-01 - 4.39e-01	52
	
	Protein trafficking	3.77e-01 - 4.39e-01	24

Linear Del	Cellular assembly and organization	1.47e-05 - 2.4e-01	75
	
	Cellular function and maintenance	1.47e-05 - 2.4e-01	44
	
	Cellular development	2.56e-02 - 2.4e-01	39
	
	Cellular growth and proliferation	2.56e-02 - 2.4e-01	15
	
	DNA replication, recombination, and repair	4.28e-02 - 2.4e-01	41

Quad Amp	Cellular development	1.57e-01 - 1.68e-01	20
	
	Cell death	1.57e-01 - 1.68e-01	25
	
	Cell cycle	1.57e-01 - 1.68e-01	16
	
	Cell morphology	1.57e-01 - 1.68e-01	24
	
	Gene expression	1.57e-01 - 1.68e-01	23

Quad Del	Post-translation modification	1.54e-01 - 2.11e-01	19
	
	Cell morphology	1.54e-01 - 2.11e-01	13
	
	Cellular development	1.54e-01 - 2.11e-01	14
	
	Carbohydrate metabolism	1.54e-01 - 2.11e-01	9
	
	Lipid metabolism	1.54e-01 - 2.11e-01	16

Canonical pathways, association network functions, disease and disorders and molecular and cellular functions were analyzed by IPA to assemble gene lists identified by linear/quadratic models for Amp and Del.

The genes with a linear relationship were involved in significant canonical pathways, including purine and pyrimidine metabolism (for both deletions and amplifications), as well as estrogen metabolism and ER signaling (linear amplification) and response to damage (linear deletion). The top canonical pathways for the genes with a quadratic relationship were specific pathways like PTEN, Neuregulin signaling and PI13K/AKT (quadratic amplification) as well as Wnt(B) and IL-2 signaling (quadratic deletion). These pathways have been very extensively studied and implied in breast cancer development. Notably, the Neuregulin pathway, containing the well-known breast cancer oncogene ERBB2, as well as PTEN and P13K/AKT signaling, was found to be a top pathway of deregulation of breast cancer at multiple levels, including DNA methylation and alelle loss. These results were obtained by a method called multiple concerted disruption (MCD) designed to identify genes aberrated at different levels. This supports our hypothesis that genes subjected to alterations in expression at multiple levels will have stronger non-linear components than genes that are merely "explained" by copy number. All four categories identified cancer as the top significant disease and cell cycle, cell signalling, and cellular development as significant networks. The linear model was more often seen with genes with overrepresentation of molecular and cellular functions of gene expression, cellular maintenance and synthesis, while genes with a nonlinear relation had functions involving development and lipid metabolism.

### Investigation for genomic locations identified by linear and quadratic models

**Figure 5:**
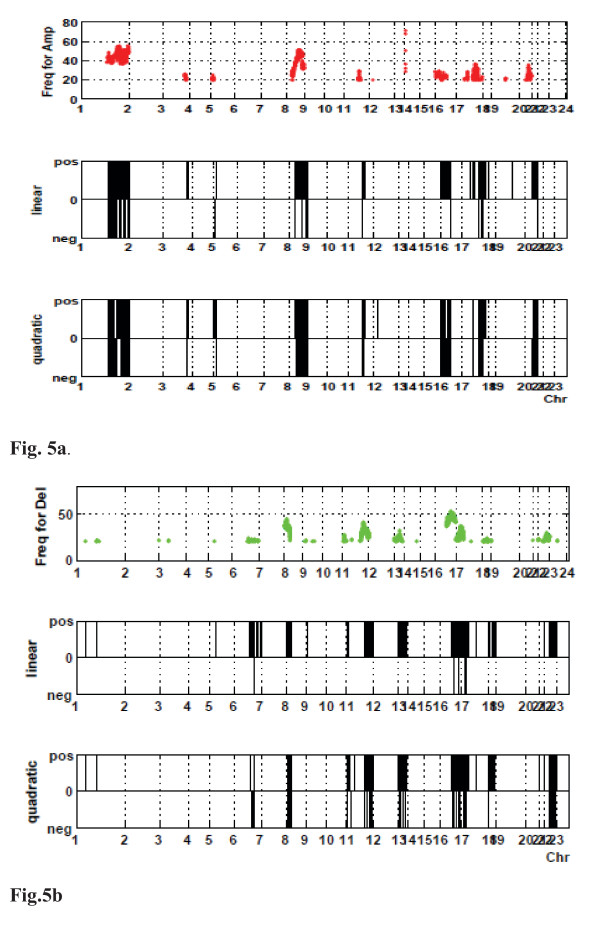
**Line maps of genomic locations for the significant DNA aberrations/mRNA expression dependences**. The line maps describe genomic locations for the significant DNA aberrations/mRNA expression dependences found by linear (top) and quadratic (middle) for Amp (a) and Del (b). The specific regions for Amp (a) and Del (b), which we detected in Methods, are illustrated in the bottom panels.

The genomic localizations of the significant DNA aberrations/mRNA expression dependences, which were found to be significant by the linear and quadratic models, were plotted on the line maps shown in Figure 5a for Amp and Figure 5b for Del. The figures present three maps: loci with linear term (top), loci with quadratic term (middle), and the loci this study is restricted to (bottom). The black lines in the maps indicate significant genomic locations obtained by these models. The vertical axis of the line maps ('positive' and 'negative') indicates whether the estimated coefficients for the linear and quadratic terms were positive or negative. These plots show that in the case of amplification (Figure 5a), the coefficients are more often positive in the loci with linear relationships but more often negative in the case of quadratic relationships. This indicates that negative regulatory mechanisms of gene expression are better explained by the quadratic model. In the case of deletion (Figure 5b), however, the opposite is observed: The relationships between DNA copy number and mRNA is more often negative in the case of linearity and positive in the case of quadratic interaction. This may indicate 'suppression' or feedback related to regulation. Interestingly, non-linear relationships were hardly ever observed on their own but mostly coincided with areas of linear relationship between DNA aberrations and mRNA expression. This finding suggests that 1) in order to become non-linear, these aberrations first need to occur, and 2) only parts of the amplicons are subjected to further transcriptional enhancement/modulation, and these peaks may point to refined target genes driving clonal selection.

### RT-PCR Validation

**Figure 6:**
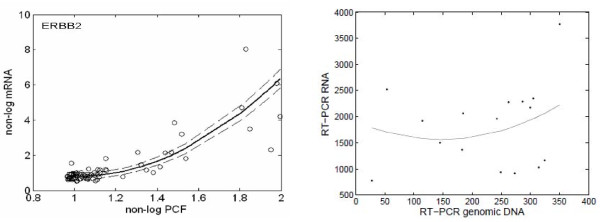
**RT-PCR Validation**. The left panel indicates ERBB2 data and quadratic predictions from array and the right panel indicates that the plots and the quadratic prediction curve of the data are given for the DNA and mRNA tumor expressed from the ERBB2 locus for TaqMan quantitative RT PCR analysis.

To address the concern that the deviation from linearity observed here may be introduced by the different dynamic range or other technical noise on the arrays (CGH and mRNA expressions), we used TaqMan to perform quantitative RT PCR analysis of the tumor DNA and mRNA expressed from the ERBB2 locus (Figure 6). The results confirmed a non-linear relationship.

## Conclusions

The statistical analysis described in this paper allowed us to distinguish linear and nonlinear dependences between copy number alterations and mRNA expression *in-cis*. We applied the procedure to 109 K logR data (Illumina) and 44 K mRNA data (Agilent) for 102 breast cancer samples and found a clear indication of deviation from linearity. This seems reasonable to expect because regulatory processes within a pathway are complex and unlikely to be subordinated to linearity. Although the linear model represents a first-order approximation of a Taylor expansion (for example of the regression function), the quality of the approximation will vary. In loci with strong nonlinearities, threshold effects and complex local interactions by a non-linear model will fit data better. However, it is difficult to draw the exact regression curve for most genes due to noise in both SNP-CGH and expression signals, in addition to inter-individual variation between the tumors. Additional single-gene-based assays will be necessary to confirm these trends obtained from large-throughput, array-based experiments. In order to explore the possibility of an even more general nature of nonlinearity, we would apply more flexible or non-parametric models rather than the simple quadratic model. It is important to consider non-linearity because CGH-mRNA correlations are used to estimate drivers in carcinogenesis, making it is essential to assess the validity of the chosen methods.

## Methods

### Study population

The study population consisted of 102 tumor samples from patients with early stage breast cancer [17]. The 44 K mRNA expression data were obtained with the Agilent platform and DNA copy number from 109 K SNP-CGH logR values were obtained using an Illumina aCGH as previously described [18]. Preprocessing of mRNA data included imputation of missing values using *LLS *in the R library called '*pcaMethod*' with k = 20. Quantile normalization was applied by *normalizeBetweenArrays *in the R library called *limma*. For the aCGH data, every tumor genome was segmented by fitting a piecewise constant function to the logR values using the Piecewise Constant Fitting (PCF) algorithm [19,20]. This algorithm produces an estimated copy number at every genomic location, including all mRNA probe locations. Accordingly, for every mRNA probe we found a corresponding copy number value. In this manner, a combined set of mRNA and logR values were obtained at 40,442 genomic locations for each of the 102 samples (raw data can be accessed from http://www.ifi.uio.no/bioinf/Projects/GenomeArchitecture).

### Linear and nonlinear modelling and its evaluation

We describe the relationship between gene expression and copy number by regression *incis*. Let *y_ij _*and *x_ij _*indicate the non-logarithmic mRNA and PCF filtered copy numbers for sample *i *and location *j*, respectively. We first describe the linear model with *i.i.d*. Gaussian noise:(1)

Note that for a copy number ratio of *x_ij _*= 1.0, the gene expression equals the intercept *α_j_*.The parameter *β_j _*quantifies the strength of the relationship between gene expression and copy number at the *j*th genomic location. Note that the interpretation of the sign of the parameter *β_j _*in models (1) and (2) will differ for Amp and Del locations. For example, a positive *β_j _*in model (1) will correspond to a positive linear relationship between expression and copy number for Amp locations and a negative relationship for Del locations.

Next, we introduce a quadratic nonlinear model represented by(2)

As for the previous model, the intercept denotes the gene expression corresponding to normal DNA copy number. Model (2) incorporates a quadratic term that allows expression to depend nonlinearly on copy number. To investigate whether a linear model fits better than the null model, we applied a *t*-test separately for amplification and deletion (*H*_0 _: *β_j _*= 0 against *H*_1_: *β_j _*≠ 0). To investigate whether a quadratic model is better than a linear model, we applied a partial *F*-test (*H*_0 _:*γ_j _*= 0 against *H*_1_: *γ_j _*≠ 0) separately for amplifications and deletions. A false discovery rate [16] was applied to correct p-values for multiple testing. In order to directly compare the model candidates, we used the Akaike information criterion (AIC) [21]. The model which gave the minimum AIC was defined to be the best AIC model. Models (1) and (2) were fitted by *robustfit *(Matlab^® ^The Mathworks http://www.mathworks.com/products/matlab).

### Categorization of genomic loci for model fitting

**Figure 7:**
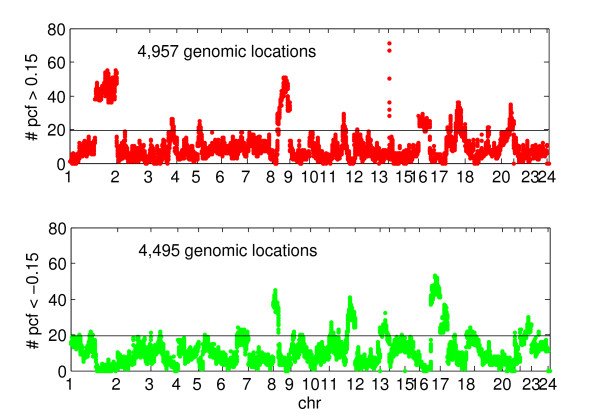
**Amp/Del genomic locations**. The vertical axis indicates the percentages of the sample number with PCF filtered values > 0.15 (red) and PCF filtered values < -0.15 (green) divided by the number of samples. The horizontal line at 20% indicates the threshold we used in this analysis. The locations above the threshold line are defined as Amp (red, 12% of all) or Del (green, 11% of all) and were then investigated for linear and nonlinear relationships, respectively.

Since amplifications and deletions in copy number aberrations may involve different regulatory mechanisms, these two events were analyzed independently. We first split the 40,442 genomic locations into categories Amp, Del and Normal, corresponding to the loci of frequent amplification, frequent deletion or neither. The Amp category consists of all genomic locations for which at least 20% of the samples had PCF-filtered (see details in Methods section) logR values above 0.15, the Del category consists of all genomic locations for which at least 20% of the PCF filtered logR values were below -0.15, and all other locations are assigned to the normal category. In theory, a location could be assigned to both Amp and Del; however, this never occurred in our data set using the above thresholds. Numbers of Amp and Del locations were 4.957 and 4.495, respectively, as described in Figure 7. For Amp locations, the linear and quadratic models were fitted to the samples with normal copy number and gain, and for Del locations the linear and quadratic models were fitted to the samples with normal copy number and loss.

Secondly, we drew a histogram of all mean values, taking the PCF fitted values for each location. We identify three peaks around the frequencies for -0.12, 0, and 0.15, and one small peak around 0.075. The central component of this mixture, representing the norm, seems to be located between -0.05 and +0.05: these samples were taken as part of both groups Amp and Del. The analyses steps are summarized in Additional file 4 and the flow diagram of the testing procedure are described in Additional file 5.

### Pathway analyses

Pathway analysis was applied in order to investigate the biological functional interaction for the lists of genes obtained by the above analysis, using a tool called Ingenuity Pathway Analysis (IPA, Ingenuity systems http://www.ingenuity.com/). This delivers a rapid assessment of the signalling and metabolic pathways, molecular networks, and biological processes that are most significantly perturbed in the dataset of interest. IPA offers many options for finding insights into relationships, mechanisms, functions and pathway of relevance.

## Authors' contributions

Biological motivation: VK and ALBD; Study design: HKS, OC and AF; PCF and data matching for CGH and mRNA: OC; Model construction and evaluation: HKS; Pathway analyses: HKS and VK; Writing of the manuscript: HKS, OC, AF, VK and ALBD. All authors have read and approved the final manuscript.

## Supplementary Material

Additional file 1 **Supplementary Figure **1**: Number of lower AIC vs number of p-values**. To assess the accuracy for the results identified by the linear and quadratic models, we compared the results related to the p-values obtained by *T *test and partial *F *test and model selection by AIC. The p-values for loci identified significantly by the quadratic model were sorted by small p-value. We calculated the top 200, 400, 600, 800, 1000 and 1811 for Amp and 200, 400, 600, 800, 1000 and 1226 for Del in the case of the linear model, and the top 100, 200, 300, 400 and 412 loci for Amp and 100, 200, 300 and 387 loci for Del in the case of the quadratic model. For each group, we also calculated AICs for the cases applying linear and quadratic models and counted the number of the cases where AIC by the quadratic model was smaller than AIC by the linear model. As can be seen in the Supplementary figures, the number of p-values was close to the number of lower AICs in most cases, indicating that the loci identified significantly by *T*-test and partial *F*-test were also significant from the viewpoint of the model selection approach based on the information criterion. Click here for file

Additional file 2 **Supplementary Figures **2a-d. The scatter plots and the predictions of the top 10 models of each of the following categories: i) linear model with positive (first five panels) and negative (latter five panels) linear coefficients for Amp (a), ii) linear model with positive and negative linear coefficients for Del (b), iii) quadratic model with positive and negative quadratic coefficients for Amp (c), and iv) quadratic model with positive and negative quadratic coefficients for Del (d). In these figures, the black dots indicate each sample that shows normal and gain (loss) regions where the model was applied, and the grey dots indicate the rest of the samples, which were distinguished as loss (gain) regions. Click here for file

Additional file 3 **Estimated coefficients, p-values, log-likelihood and AIC in top 10 linear and quadratic models corresponding to Additional file **2** Supplementary Figure **2a-d**are summarized**. Click here for file

Additional file 4 **The analyses steps**. Click here for file

Additional file 5 **Flow diagram of the testing procedure**. Click here for file
