# Supplementary material for: Linear and non-linear dependencies between copy number aberrations and mRNA expression reveal distinct molecular pathways in breast cancer
Source: BMC Bioinformatics. 2011 May 24;12:197. doi: 10.1186/1471-2105-12-197 (PMC3128865; doi:10.1186/1471-2105-12-197)
Supplement: Additional file 1 — Supplementary Figure 1: Number of lower AIC vs number of p-values. To assess the accuracy for the results identified by the linear and quadratic models, we compared the results related to the p-values obtained by T test and partial F test and model selection by AIC. The p-values for loci identified significantly by the quadratic model were sorted by small p-value. We calculated the top 200, 400, 600, 800, 1000 and 1811 for Amp and 200, 400, 600, 800, 1000 and 1226 for Del in the case of the linear model, and the top 100, 200, 300, 400 and 412 loci for Amp and 100, 200, 300 and 387 loci for Del in the case of the quadratic model. For each group, we also calculated AICs for the cases applying linear and quadratic models and counted the number of the cases where AIC by the quadratic model was smaller than AIC by the linear model. As can be seen in the Supplementary figures, the number of p-values was close to the number of lower AICs in most cases, indicating that the loci identified significantly by T-test and partial F-test were also significant from the viewpoint of the model selection approach based on the information criterion. [file 1471-2105-12-197-S1.PDF]

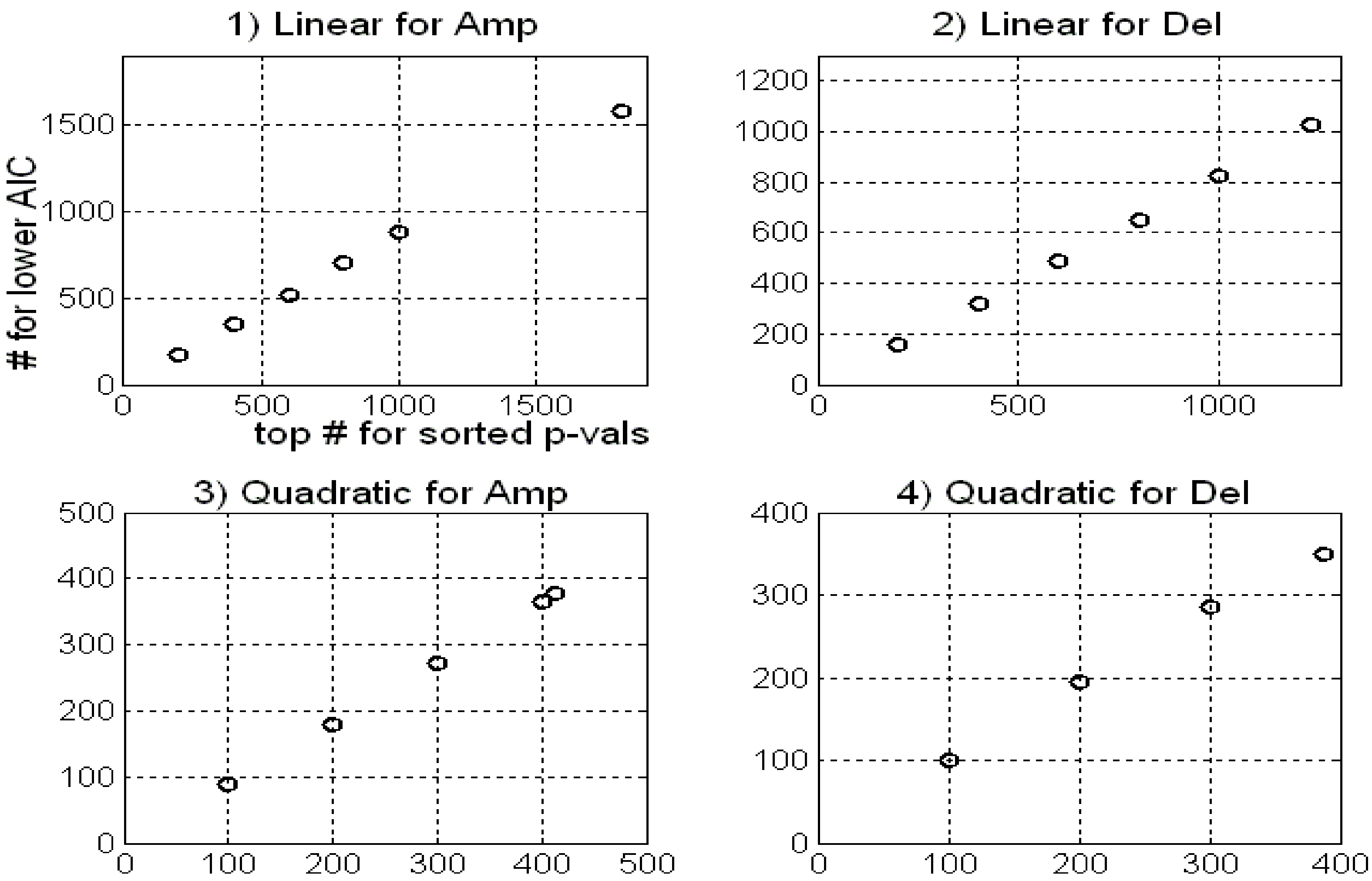

To assess the accuracy for the results identified by the linear and quadratic models, we compared the results related to the p-values obtained by *T* test and partial *F* test and model selection by AIC. The p-values for loci identified significantly by the quadratic model were sorted by small p-value. The top 200, 400, 600, 800, 1000 and 1811 for Amp and 200, 400, 600, 800, 1000 and 1226 for Del in the case of linear model, and the top 100, 200, 300, 400 and 412 loci for Amp and 100, 200, 300 and 387 loci for Del in the case of quadratic model were calculated. For each group, we also calculated AICs for the cases applying linear and quadratic models and counted the number of the cases that AIC by the quadratic model was smaller than AIC by the linear model. As can be seen in figures 1) – 4) , the number of p-values were close to the number of lower AICs in most of the cases, indicating that the loci identified significantly by *T*-test and partial *F*-test were also significant from the view of model selection approach based on the information criterion.

Supplementary Figure 1.
