# Supplementary material for: Linear and non-linear dependencies between copy number aberrations and mRNA expression reveal distinct molecular pathways in breast cancer
Source: BMC Bioinformatics. 2011 May 24;12:197. doi: 10.1186/1471-2105-12-197 (PMC3128865; doi:10.1186/1471-2105-12-197)
Supplement: Additional file 2 — Supplementary Figures 2a-d. The scatter plots and the predictions of the top 10 models of each of the following categories: i) linear model with positive (first five panels) and negative (latter five panels) linear coefficients for Amp (a), ii) linear model with positive and negative linear coefficients for Del (b), iii) quadratic model with positive and negative quadratic coefficients for Amp (c), and iv) quadratic model with positive and negative quadratic coefficients for Del (d). In these figures, the black dots indicate each sample that shows normal and gain (loss) regions where the model was applied, and the grey dots indicate the rest of the samples, which were distinguished as loss (gain) regions. [file 1471-2105-12-197-S2.PDF]

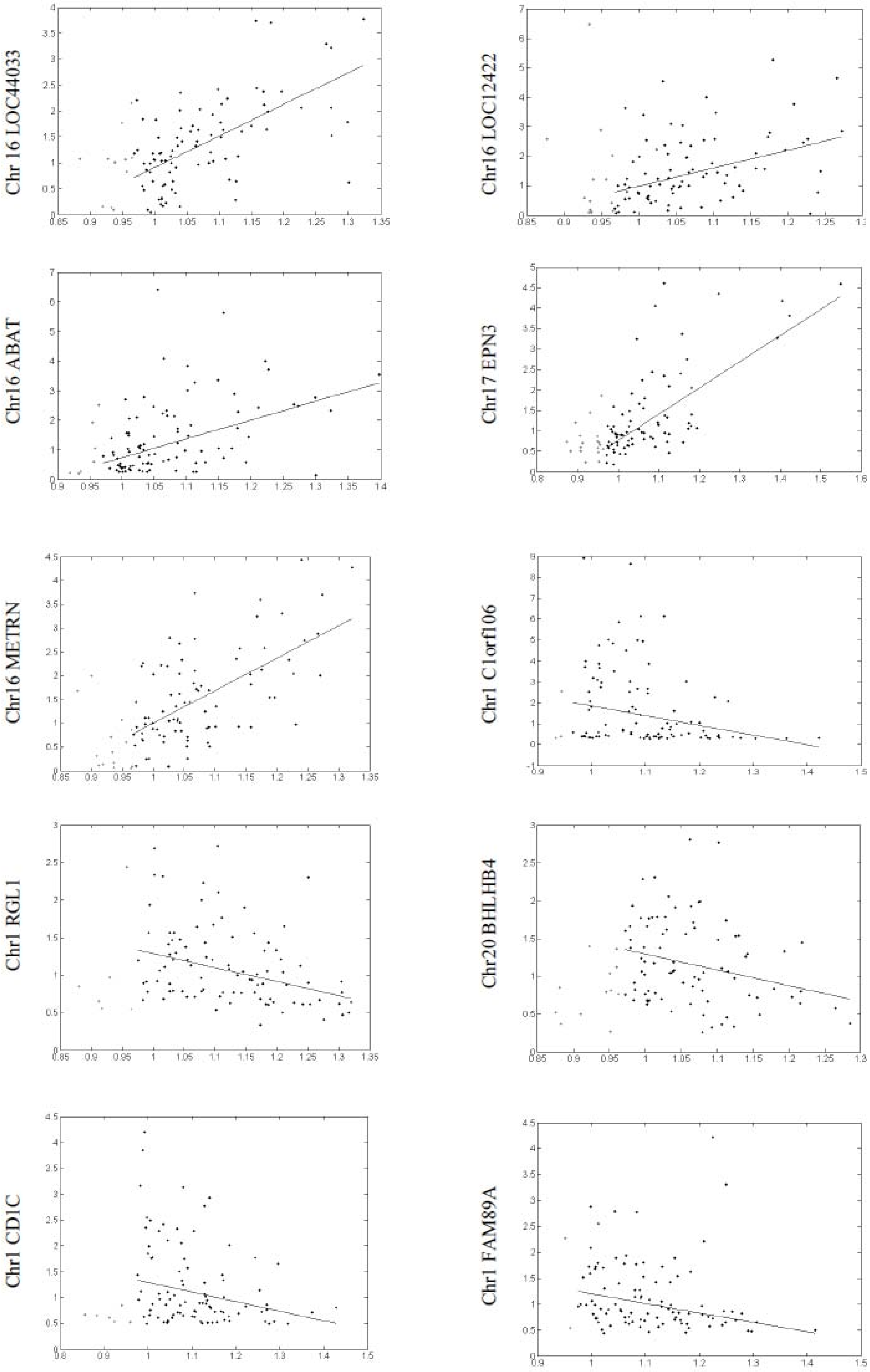

Top10 significant linear models for Amp

Supplementary Figure 2 a

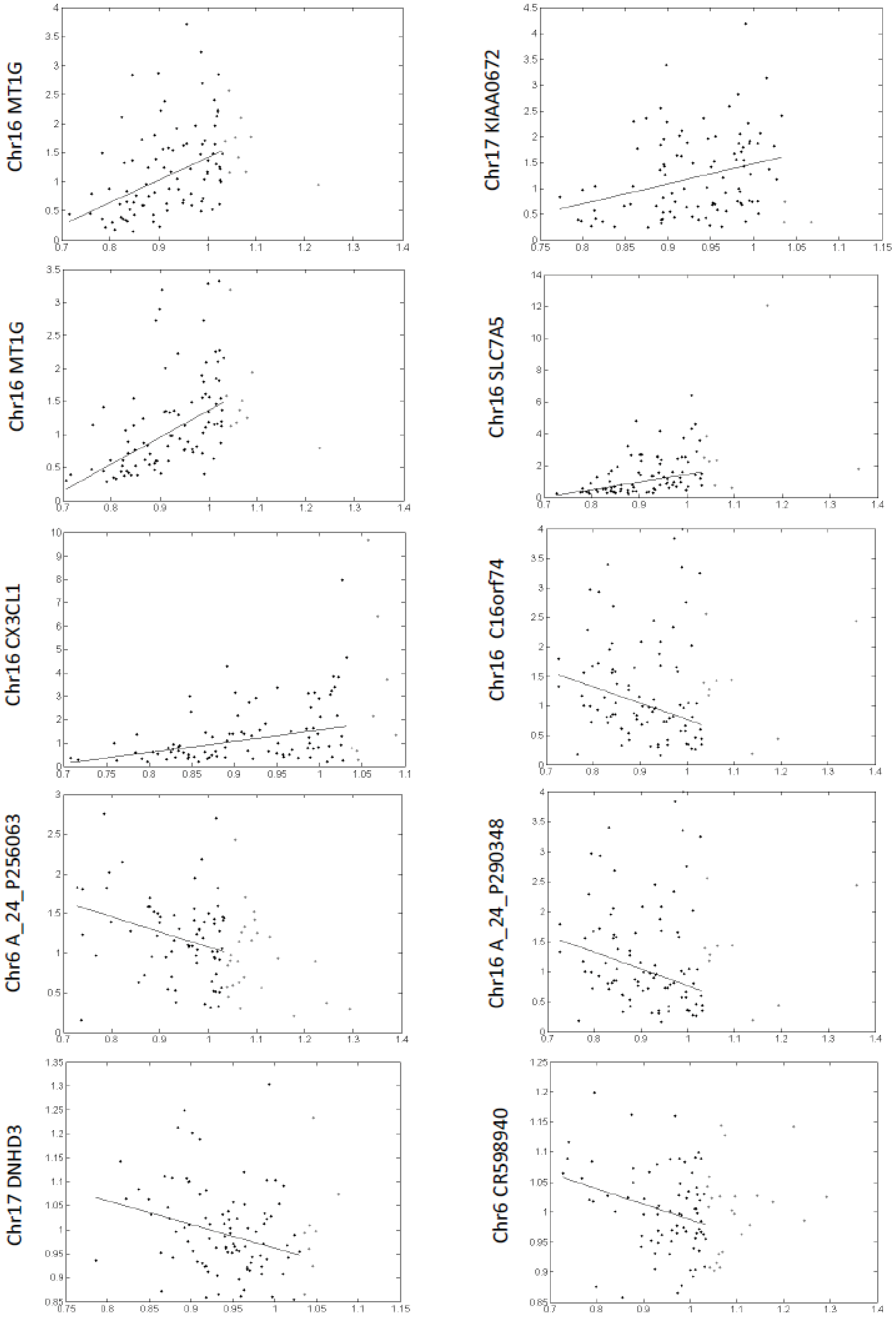

Top 10 significant linear models for Del

Supplementary Figure 2 b

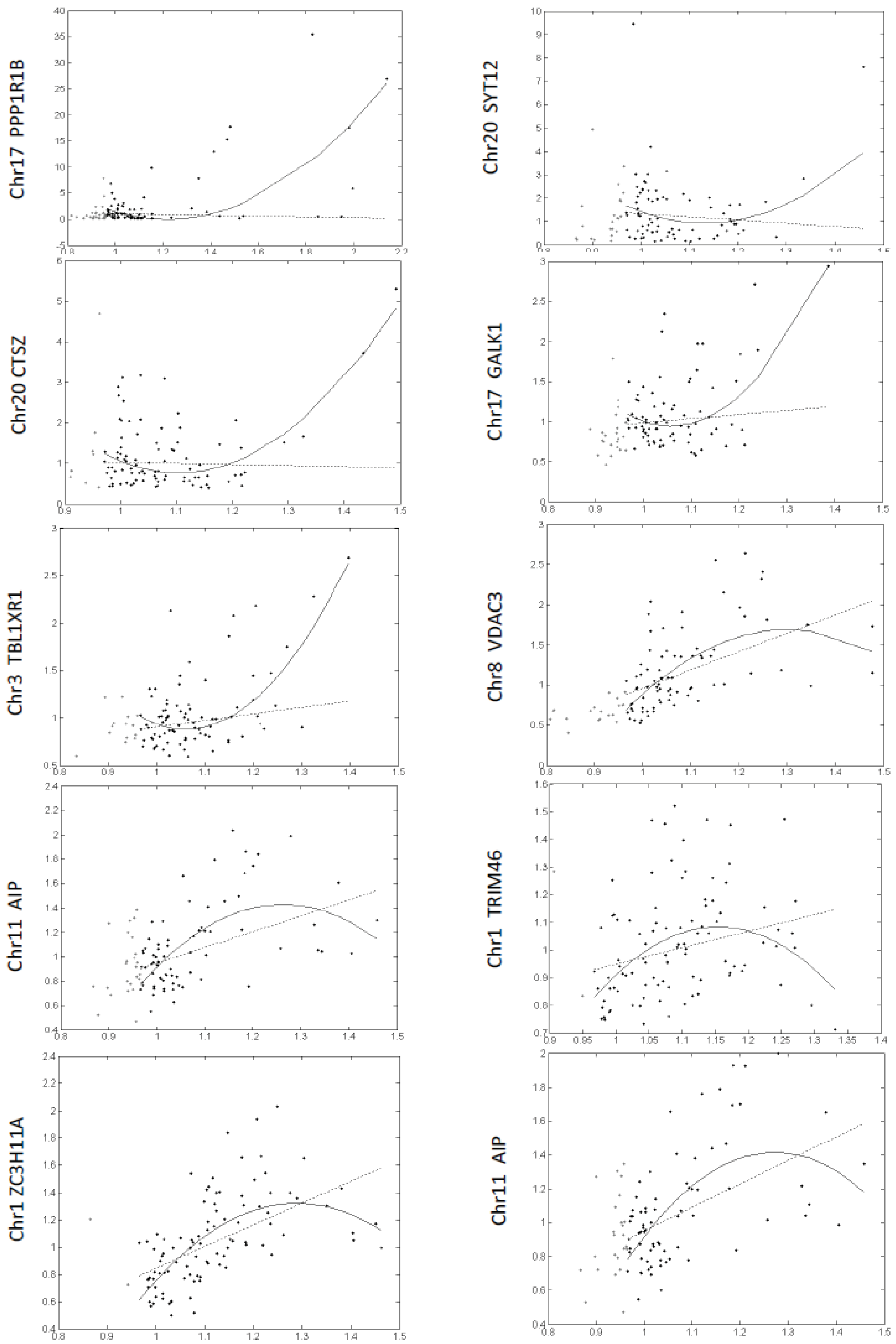

Top 10 significant quadratic models for Amp

Supplementary Figure 2 c

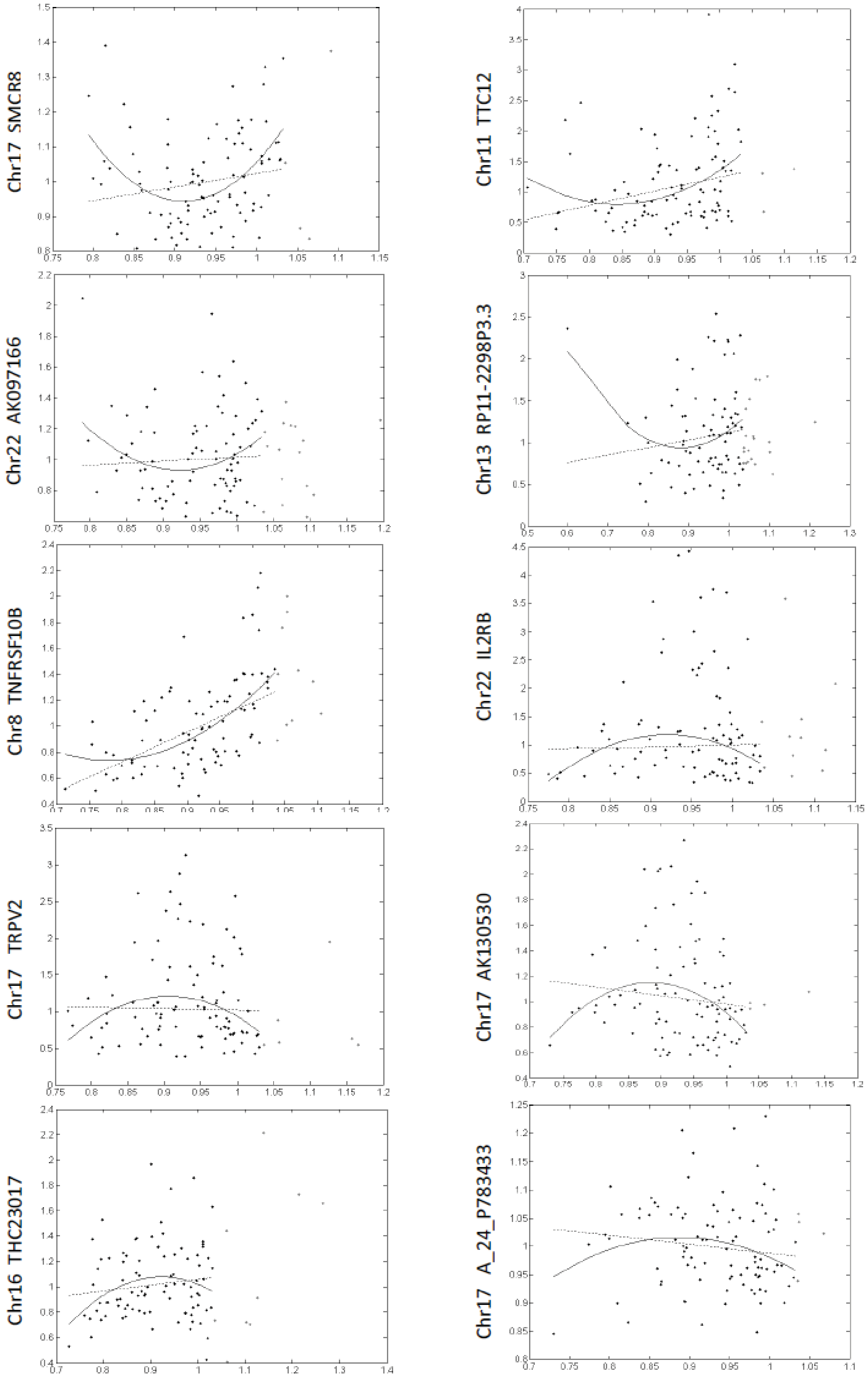

Top 10 significant quadratic models for Del

Supplementary Figure 2 d
