# Supplementary material for: Linear and non-linear dependencies between copy number aberrations and mRNA expression reveal distinct molecular pathways in breast cancer
Source: BMC Bioinformatics. 2011 May 24;12:197. doi: 10.1186/1471-2105-12-197 (PMC3128865; doi:10.1186/1471-2105-12-197)
Supplement: Additional file 4 — The analyses steps. [file 1471-2105-12-197-S4.PDF]

1. Apply PCF to logR for all genomic locations and transform them to PCF-fitted smoothing values.

2. Specify Amp/Del genomic locations for PCF-fitted values with the thresholds -0.15/0.15. This allows the inclusion of as many as 80% of the samples for each location.

3. To apply the models for each location in Amp/Del, take samples related to gain/loss and the normal copy number for > -0.05 / < 0.05.

4. Apply linear and quadratic models to the relative amounts of Amp and Del with (PCF-fitted value – 1.0) and (1.0 – PCF-fitted value) for each sample and estimate the coefficients by *robustfit* (Matlab®) to avoid outliers.

5. Apply *T*-test for linear model and partial *F*-test for quadratic model. Use AIC to select the best-fit model for each location in Amp/Del.

6. Assign certain gene symbols to the locations that indicate significant linear/quadratic relationships and then list them.

7. Apply pathway analysis to the gene lists.
