# Supplementary figures and images for: Linear and non-linear dependencies between copy number aberrations and mRNA expression reveal distinct molecular pathways in breast cancer
Source: BMC Bioinformatics. 2011 May 24;12:197. doi: 10.1186/1471-2105-12-197 (PMC3128865; doi:10.1186/1471-2105-12-197)

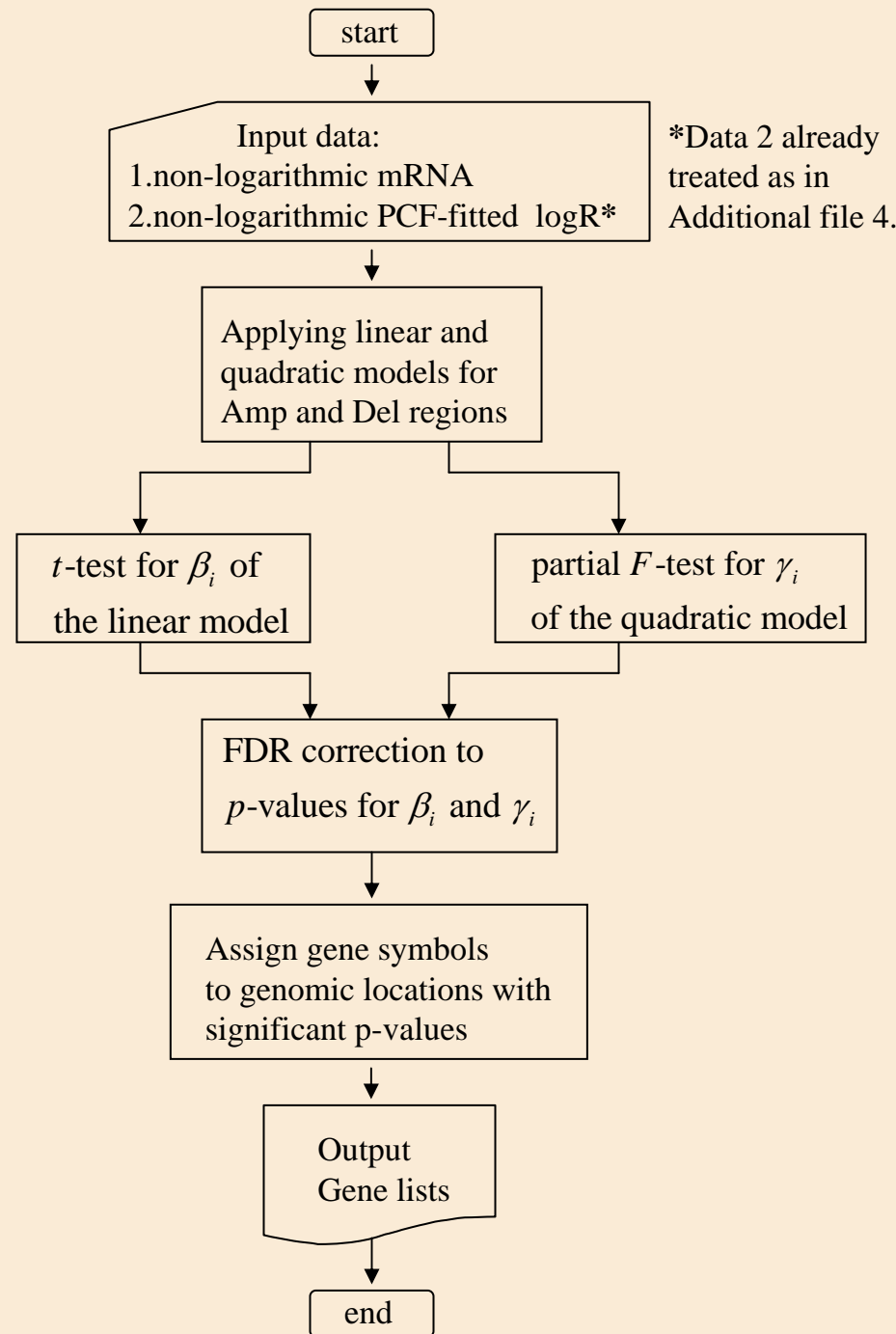

Supplement: Additional file 5 — Flow diagram of the testing procedure. [file 1471-2105-12-197-S5.PDF]
